# Iterative Robust Capon Beamforming with Adaptively Updated Array Steering Vector Mismatch Levels

**DOI:** 10.1155/2014/260875

**Published:** 2014-11-03

**Authors:** Tao Zhang, Liguo Sun

**Affiliations:** Department of Electronic Engineering and Information Science, University of Science and Technology of China, Hefei 230027, China

## Abstract

The performance of the conventional adaptive beamformer is sensitive to the array steering vector (ASV) mismatch. And the output signal-to interference and noise ratio (SINR) suffers deterioration, especially in the presence of large direction of arrival (DOA) error. To improve the robustness of traditional approach, we propose a new approach to iteratively search the ASV of the desired signal based on the robust capon beamformer (RCB) with adaptively updated uncertainty levels, which are derived in the form of quadratically constrained quadratic programming (QCQP) problem based on the subspace projection theory. The estimated levels in this iterative beamformer present the trend of decreasing. Additionally, other array imperfections also degrade the performance of beamformer in practice. To cover several kinds of mismatches together, the adaptive flat ellipsoid models are introduced in our method as tight as possible. In the simulations, our beamformer is compared with other methods and its excellent performance is demonstrated via the numerical examples.

## 1. Introduction

The adaptive beamforming has found wide applications in many aspects, such as radar, sonar, biomedicine, radio astronomy, and wireless communication. With loss of generalization, adaptive beamforming aims to adjust the main lobe of array beam pattern to focus on the direction of signal of interest (SOI) and suppress the interference and noise simultaneously. In the design of the traditional beamformers, the exact prior information about the ASV is demanded. For example, the MVDR beamformer [1] sets a distortionless constraint on the SOI to maximize the output SINR. And yet, when there was a mismatch between the presumed DOA of SOI and the assumed one, caused by the array mismatches, the array undergoes performance deterioration. However, many array imperfections, like the surrounding environment fluctuation, multipath, coupling, and others [2–5], cannot be ignored in practice. As a result, the DOA of SOI is distributed in an uncertainty region instead of a precise point. Therefore, robust adaptive beamforming (RAB) has been an attractive research topic and many approaches are reported in [1, 6–16]. And these methods can be broadly divided into two groups: the linearly constrained [1, 6, 7] and quadratically constrained beamformers [11–16].

The linearly constrained robust adaptive beamforming (LC_RAB) algorithms [1, 6, 7] impose several linear magnitude constraints to force the array response to be unity in the uncertainty region of look direction and then the main beam of array pattern is broadened to cover the uncertainty set absolutely. Nevertheless, the array suffers resolution degradation and the response ripples in the main beam. To overcome the disadvantages of LC_RAB, the robust capon beamformer (RCB) [12, 13] is proposed with a spherical uncertainty set about the ASV of SOI. The center of the set is the presumed ASV and the radius of the sphere is the norm of the mismatch ASV between the presumed ASV and the actual ASV. And the upper bound of radius is assumed to be known. In [14], the authors impose an extra constant norm constraint on the ASV and this beamformer is referred to as the doubly constrained RCB (DCRCB). It is found that the RCB and DCRCB are all belonging to the diagonal loading (DL) method [10]. The optimal loading factors in these beamformers can be derived on the basis of current mismatch level, while the loading factor in [10] is selected by the designers themselves. Unfortunately, neither the mismatch vector nor the upper bound of its radius is known. If the upper bound is underdetermined, the self-nulling of the SOI may emerge and when the large bound occurs, the output SINR of the RCB and DCRCB experience degradation. Different from the [11–16], Nai et al. introduce a series of beamfomers to search the actual ASV iteratively until the stopping condition is satisfied; each iteration is based upon the RCB using fixed and smaller uncertainty level to achieve the high output SINR [17, 18]. At each step, the current presumed ASV is updated by the normalized estimated ASV of the previous step to avoid the scaling ambiguity. However, the optimal error levels in these methods are also unknown.

To maintain the output performance and obtain the optimal error radius, we develop an iterative RCB with adjustable uncertainty levels (Au-IRCB). In this method, the notion of iterative robust adaptive beamforming (IRAB) is reconstructed and the radius of uncertainty sets is adjusted adaptively at each step. Here, we use the traditional subspace decomposition theory to construct the estimation of mismatch vectors through a convex optimal equation. According to the subspace decomposition theory [8, 9], two orthogonal subspaces, denoted as signal-interference subspace (SIS) and noise subspace, respectively, can be deduced from the eigendecomposition of sample data variance matrix. The ASV of SOI lies in the SIS and its projection onto the noise approaches zero. Based on this characteristic, the norm of the projection vectors onto the SIS equals the square root of the elements number. we make use of this norm constrain to calculate optimal radius of the uncertainty set; this suboptimal equation is in the form of quadratic programming quadratically constrained problem (QCQP) [19]. In this case, the estimated error radiuses in the initial iterations are large in accord with the error between the initially presumed ASV and actual ASV. Then the subsequent radiuses decrease along with the convergence. On the other hand, the estimated error levels are smaller than the mismatch level used in [12, 13]; then the output SINR is retained. In addition, some other defections, like array elements position, interelement and coupling et al. [2–5], should be taken into account in the modeling process [13, 15, 20, 21]. Instead of the sphere uncertainty set, the minimum flat ellipsoid is calculated in [13, 15], to model the ASV of SOI in the presence of multiple errors. Similarly, we also test the Au-IRCB with adjustable ellipsoid set to tackle with the large look direction error and array element position displacement together. Finally, the experiment results prove the correctness of our theory and the Au-IRCB provides more robustness than common beamformers in the more severe simulation environment.

## 2. Robust Capon Beamforming

Consider *K* signals impinge on the array of *M* isotropic elements. The narrow-band received data *X*(*t*) ∈ *C* ^*M*×1^, at time *t*, can be expressed as

(1) $$\begin{array}{c} X\left(t\right)=X_{s}\left(t\right)+X_{i}\left(t\right)+n\left(t\right), \end{array}$$

where *X*_*s*_(*t*), *X*_*i*_(*t*), and *n*(*t*) denote the vectors of desired signal, interference, and noise, respectively. All the signal, interference, and noise components in (1) are assumed to be statistically independent of each other. The *a*_*s*_ is the ASV of the desired signal, and the desired signal component can be expressed as *X*_*s*_(*t*) = *s*(*t*)*a*_*s*_. The output of beamformer is shown by

(2) $$\begin{array}{c} y\left(t\right)=w^{H}x\left(t\right), \end{array}$$

where *w* is the *M* × 1 complex weight vector and (·)^*T*^ and (·)^*H*^ stand for transpose and Hermitian transpose, respectively [1]. Then, the output SINR is displayed as

(3) $$\begin{array}{c} \text{SNIR}=\frac{P_{1}{\left|w^{H}a_{s}\right|}^{2}}{w^{H}R_{\text{in}}w}, \end{array}$$

where *R*_in_ is the interference and noise covariance matrix ∈*C* ^*M*×*M*^ and *P*_1_ = *δ*_*s*_^2^ is the power of desired signal:

(4) $$\begin{array}{c} R_{\text{in}}=E\left\{{\left(\overset{K}{\underset{i=2}{\sum }}a_{i}s_{i}+n(t)\right)}^{H}\left(\overset{K}{\underset{i=2}{\sum }}a_{i}s_{i}+n\left(t\right)\right)\right\}. \end{array}$$

Since the *R*_in_ is not readily available in practice, it is substituted by the sample covariance matrix

(5) $$\begin{array}{c} \hat{R}=\frac{1}{N}\overset{K}{\underset{i=1}{\sum }}x\left(i\right)x^{H}\left(i\right), \end{array}$$

where *N* is the number of training data samples. It is easy to obtain the optimal weight vector by means of the distortionless response toward the desired signal and maximizing the output SINR. Hence, the maximization of (3) can be written as

(6) min⁡w wHR^ws.t.     wHas=1.

The above equation is known as MVDR. The optimal solution of (5) can be easily found, $w_{\text{opt}}=\beta {\hat{R}}^{-1}a_{s}$, where $\beta ={\left(a_{s}^{H}{\hat{R}}^{-1}a_{s}\right)}^{-1}$ is a constant and it has no influence on the SINR. Then, substituting *w*_opt_ into (3), one yields the maximum of output SINR:

(7) $$\begin{array}{c} {\text{SINR}}_{\text{opt}}=P_{1}a_{s}^{H}{\hat{R}}^{-1}a_{s}. \end{array}$$

The MVDR beamformer is known to be dependent on the precisely prior information of the ASV of SOI. As a consequence, it does not provide sufficient robustness against the ASV mismatches caused by multiple array imperfections. When there is a mismatch between the presumed ASV $\bar{a}$ and the actual ASV *a*, an uncertainty set is chosen to be a sphere $\left\{a,{\left\|a-\bar{a}\right\|}^{2}\leq \varepsilon \right\}$, where $\bar{a}$ and *ε* are the presumed ASV and upper bounded of the error level, respectively. The problem (6) belongs to the conventional RCB in the case of *ε* = 0:

(8) min⁡a aHR^−1as.t.    a−a¯≤ε.

This optimization equation can be solved and the solution is given by

(9) $$\begin{array}{c} {\hat{a}}_{0}=\bar{a}-{\left(I+\lambda \hat{R}\right)}^{-1}\bar{a} \end{array}$$

and the Lagrange multiplier *λ* is determined by the constraint

(10) $$\begin{array}{c} {\left\|{\left(I+\lambda \hat{R}\right)}^{-1}\bar{a}\right\|}^{2}=\varepsilon . \end{array}$$

When the uncertainty set is flat ellipsoid, the RCB with ellipsoid set [15] is described as

(11) min⁡a a¯+PuHR^−1a¯+Pus.t.    a=a¯+Pu, u≤1,

where *P* is a *M* × *L*  (*L* < *M*) matrix and *u* is a *L* × 1 vector. Let $\tilde{R}=P^{H}R^{-1}P$ and $\bar{\tilde{a}}=P^{H}R^{-1}\bar{a}$. The Lagrange can also be used to obtain the optimal solution $\hat{u}=-{\left(\tilde{R}+\tilde{\lambda }I\right)}^{-1}\bar{\tilde{a}}$ and the $\tilde{\lambda }$ is the unique solution of equation

(12) $$\begin{array}{c} {\left\|{\left(\tilde{R}+\lambda I\right)}^{-1}\overset{-}{\tilde{a}}\right\|}^{2}=1. \end{array}$$

Then the estimated ASV is given as $\tilde{a}=\bar{a}+P\hat{u}$.

## 3. Subspace Projection Theory

The eigendecomposition of sample covariance matrix is written as $\hat{R}=\sum_{i=1}^{M}\lambda_{i}e_{i}e_{i}^{H}$, where *e*_*i*_, *i* = 1,2,…, *M*, are the eigenvectors and *λ*_1_ ≥ *λ*_2_ ≥ ⋯≥*λ*_*K*_ > *λ*_*k*+1_ = ⋯*λ*_*M*_ = *δ*_*n*_^2^ are the corresponding eigenvalues, ordered in descending order. By splitting the eigenvalues into *K* largest eigenvalues and *M* − *K* smallest ones, $\hat{R}$ is rewritten as

(13) $$\begin{array}{c} \hat{R}=E_{s}\Lambda_{s}E_{s}^{H}+E_{n}\Lambda_{n}E_{n}^{H}, \end{array}$$

where *E*_*s*_ = [*e*_1_,…, *e*_*K*_] and *E*_*n*_ = [*e*_*K*+1_,…, *e*_*M*_], and the diagonal elements of the matrix Λ_*s*_ are the *K* largest eigenvalues and the *M* − *K* smallest ones are in the Λ_*n*_, respectively [8, 9]. According to the subspace decomposition theory, the signal-interference subspace is spanned by the columns of *E*_*s*_, and it is orthogonal to noise subspace, spanned by the *E*_*n*_. Let *a*_*s*/*E*_*n*__ and *a*_*s*/*E*_*s*__ denote the projection vector of *a*_*s*_ onto the noise subspace and signal-interference subspace, respectively. Hence, the *a*_*s*_ is the sum of *a*_*s*/*E*_*n*__ and *a*_*s*/*E*_*s*__, *a*_*s*_ = *a*_*s*/*E*_*s*__ + *a*_*s*/*E*_*n*__. Therefore, the projection of actual ASV *a*_*s*_ onto the noise subspace approximates zero vector. The proof is shown below:

(14) $$\begin{array}{c} a_{s/E_{n}}=E_{n}E_{n}^{H}a_{s}=E_{n}\overset{K}{\underset{i=1}{\sum }}c_{i}E_{n}^{H}e_{i},\;\;a_{s}=\overset{K}{\underset{i=1}{\sum }}c_{i}e_{i}, \end{array}$$

where *c*_*i*_  (1 ≤ *i* ≤ *K*) is real constant. For the orthogonality between *e*_1≤*i*≤*K*_ and *e*_*K*+1≤*j*≤*M*_, it can be concluded that ‖*a*_*s*/*E*_*n*__‖^2^ = 0. This deduction can be applied to construct the estimator of ASV mismatch levels; the details are summarized in Section 4.

## 4. The Proposed Algorithm

### 4.1. The Estimation of Mismatch Level

Here, we consider the error vector as $\vec{e}=a-\bar{a}$. The condition (14) can be used as a constrained condition in the following optimal equation to estimate the minimum of mismatch level ${\left\|\vec{e}\right\|}_{\min}^{2}$:

(15) min⁡a a−a¯2s.t.    EnEnHa2=0.

In this case, the Lagrange multiplier *λ* = 0 and ${\left\|\vec{e}\right\|}_{\min}^{2}$ is always zero. As a result, this estimation is insignificant. To exclude the trivial solution ${\left\|\vec{e}\right\|}_{\min}^{2}=0$, a relaxation *S* = {*a*, ‖*E*_*n*_ *E*_*n*_^*H*^ *a*‖ ≤ *δ*} is induced, and we assign a relatively small value to the *δ* and then (14) is reformulated to

(16) min⁡a a−a¯2s.t.    EnEnHa2≤δ.

The problem (16) is a typical convex problem [19] and it has sole solution. By considering the Lagrange function, we get

(17) $$\begin{array}{c} g\left(a,\lambda \right)={\left\|a-\bar{a}\right\|}^{2}+\lambda \left(\left\|E_{n}E_{n}^{H}a\right\|-\delta \right), \end{array}$$

where *λ* ≥ 0 is real-valued Lagrange multiplier. The minimizer of *g*(*a*, *λ*) for *λ* is obtained through the differentiation of (10) with respect to *a*:

(18) $$\begin{array}{c} \left(a-\bar{a}\right)+\lambda E_{n}E_{n}^{H}a=0. \end{array}$$

Equation (18) yields

(19) $$\begin{array}{c} \hat{a}={\left(I+\lambda E_{n}E_{n}^{H}\right)}^{-1}\bar{a}. \end{array}$$

Here, (19) can be simplified with the assistance of matrix inverse lemma (*I* + *AB*)^−1^ = *I* − *A*(*I* + *BA*)^−1^ *B*:

(20) $$\begin{array}{c} \hat{a}=\left(I-\frac{\lambda }{1+\lambda }E_{n}E_{n}^{H}\right)\bar{a}. \end{array}$$

It is noted that $\hat{a}$ exists only on the boundary of *S* with the evidence deduced as follows. Firstly, an assumption is given that $\bar{a}$ satisfies the norm constraint ${\left\|\bar{a}\right\|}^{2}=M$. Then the objective function is reformed as

(21) a−a¯2=a2−2Rea¯Ha+M.

When *a* is scaled by any factor *β*  (0 < *β* < 1) and the *βa* is also belonging to the *S* based on the characteristic of convex set [19], (21) is rewritten as

(22) βa−a¯2=β2a2−2βRea¯Ha+M=ββa2−2Rea¯Ha+M<a2−2Rea¯Ha+M=a−a¯2.

Based on the (22), we can see that if the optimal ASV lies inside the set *S*, the minimum mismatch will approach zero for *β* → 0. Therefore, the feasible region of this problem can only be the boundary of *S*:

(23) $$\begin{array}{c} {\left\|E_{n}E_{n}^{H}a\right\|}^{2}=\delta . \end{array}$$

Then, inserting (20) into the (23),

(24) $$\begin{array}{c} {\left\|E_{n}E_{n}^{H}\hat{a}\right\|}^{2}={\left\|\frac{1}{1+\lambda }E_{n}E_{n}^{H}\bar{a}\right\|}^{2}=\delta . \end{array}$$

We find

(25) $$\begin{array}{c} \hat{\lambda }=\sqrt{\frac{{\bar{a}}^{H}E_{n}E_{n}^{H}\bar{a}}{\delta }}-1. \end{array}$$

Once the Lagrange multiplier $\hat{\lambda }$ is given, $\hat{a}$ is determined by (19) as well as the mismatch level:

(26) $$\begin{array}{c} \varepsilon_{0}={\left\|\hat{\vec{e}}\right\|}_{\min}^{2}={\left\|\hat{a}-\bar{a}\right\|}^{2}={\left\|\frac{\hat{\lambda }}{1+\hat{\lambda }}E_{n}E_{n}^{H}\bar{a}\right\|}^{2}=\gamma^{2}{\left\|E_{n}E_{n}^{H}\bar{a}\right\|}^{2}, \end{array}$$

where *γ* is a scalar, and the mismatch vector $\vec{e}$ is the scaled projection vector of the presumed ASV onto the noise subspace:

(27) $$\begin{array}{c} \hat{\vec{e}}=rE_{n}E_{n}^{H}\bar{a},\;\;\gamma =\frac{\hat{\lambda }}{1+\hat{\lambda }}. \end{array}$$

The estimated *ε*_0_ is the minimum mismatch level between $\bar{a}$ and *a*_*s*_, and the optimal error is not equal to *ε*_0_. Obviously, an inequation must be taken into account, *ε*_opt_ ≥ *ε*_0_, and *ε*_opt_ = *ε*_0_ + Δ_*ε*_, where Δ_*ε*_ is the estimation error. In order to modify the difference between the estimated result and actual mismatch, the additional estimations are necessary. Finally, we extend this idea and propose the iterative robust beamformer with adaptively updated uncertainty levels. Similarly, Lie et al. propose another IRCB with adjustable error radiuses [22]. However, we find that there is a controversial analysis. The uncertainty radius in [22] is calculated based on the assumption that the projection of presumed ASV onto the signal subspace is collinear with the actual ASV. This geometrical approximation appears to be simple, but it may not be generalized beyond the specific context.

### 4.2. Robust RCB with Adaptive Sphere Uncertainty Sets

Based on the closed-form solution of error level (26), we can further the conventional RCB with estimated uncertainty level. What calls for special attention is that the estimated ASV just gets more close to the actual ASV than the presumed ASV. To search the ASV of the desired signal, the additional estimations of mismatch between the current presumed ASV and actual ASV are needed in occurrence of Δ_*ε*_ ≠ 0. In this way, the radius of uncertainty set used in each step is adaptively updated on the basis of a specific principle. It can be easily found that the estimated levels drop gradually. This concept is defined as iterative RCB with adjustable uncertainty radiuses.

We denote *ε*_*g*_^*i*^ as uncertainty level in *i*th iteration. From the analysis discussed above, *ε*_*g*_^*i*^ is adjusted in line with the current mismatch amount so that the initial search progress converges faster than latter steps for the diminishing uncertainty radius as the estimated vectors approach the actual ASV. To eliminate the scaling ambiguity, ${\hat{a}}_{i}$ is replaced by $\sqrt{M}{\hat{a}}_{i}/\left\|{\hat{a}}_{i}\right\|$. Again, the scaled ${\hat{a}}_{i}$ is imposed to be center of spherical uncertainty set in the next iteration to solve *ε*_*g*_^*i*+1^. The search process is continued in similar way until the desired ASV is reached. It is well known that a decision condition is necessary to terminate the iteration at optimal point. Here, we set the stopping condition as

(28) $$\begin{array}{c} \varepsilon_{g}^{i}\leq \zeta . \end{array}$$

**Figure 1:**
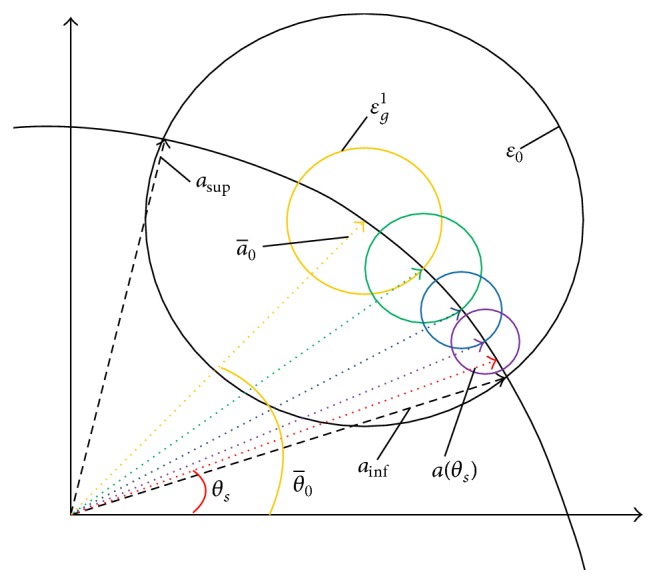
Generalized convergence trajectory of the Au-IRCB.

The *ε*_*g*_^*i*^ approaches zero at convergent. Therefore, *ζ* is selected with a very small value. For *i*th (*i* ≥ 2) iteration, the error level is set to be $\varepsilon_{g}^{i}=\gamma_{i}{\left\|E_{n}E_{n}^{H}{\hat{a}}_{i-1}\right\|}^{2}$, where ${\hat{a}}_{i-1}$ is the calculated ASV of the previous iteration.  Figure 1 illustrates the concept of AU-IRCB. When there is a DOA error Δ_*θ*_, the direction of SOI is distributed uniformly in an uncertainty region $\left[\begin{array}{cc} {\bar{\theta }}_{0}-\Delta_{\theta }/2 & {\bar{\theta }}_{0}+\Delta_{\theta }/2 \end{array}\right]$, where ${\bar{\theta }}_{0}$ are the presumed direction of SOI in the center of uncertainty region. The boundaries ASVs *a*_sup⁡_ and *a*_inf⁡_ (corresponding to the direction of ${\bar{\theta }}_{0}-\Delta \theta /2$ and ${\bar{\theta }}_{0}+\Delta \theta /2$, resp.) do not coincide with the presumed ASV (corresponding to the presumed direction ${\bar{\theta }}_{0}$). In the conventional RCB, the uncertainty level *ε* is large. Differently, the smaller radiuses are used in the proposed method (the colorized spheres). It can be seen that the radius of spheres tends to decrease progressively along with the convergence. When the estimated ASV reaches the actual ASV *a*(*θ*_*s*_) (*θ*_*s*_ is the direction of desired signal), the iteration is ended.

### 4.3. Iterative RCB with Adaptive Ellipsoid Uncertainty Sets

Several flat ellipsoid uncertainty sets have been reported in practice; there are other imperfections except the DOA error, while the spherical uncertainty cannot cover other ASV mismatches together. Then, the ellipsoid set is considered to solve the optimal ASV in the presence of multiple array errors. Recently, several flat ellipsoid uncertainty sets have been reported [13, 15] as tight as possible. Lorenz and Boyd incorporate a type model of flat ellipsoid with the aid of some prior information [15]:

(29) $$\begin{array}{c} \Omega =\left\{Pu+\bar{a},\left\|u\right\|\leq 1\right\}, \end{array}$$

where $\bar{a}\in C^{M\times 1}$ is the center of ellipsoid, *u* is *L* × 1 vector, and *P* ∈ *C* ^*M*×*L*^ delineates the geometrical shape. To extend the application range of AU-IRCB, we use the adaptive ellipsoid sets in the research process through adjusting the matrix *P*. For any vector *a*_*Ω*_ ∈ *Ω*, it satisfies $a_{\Omega }-\bar{a}=Pu$. This conclusion infers that the error vector is the linear combination of the columns of *P*. In [13], Li et al. give $P_{\text{small}}=\left[\begin{array}{cc} a\left({\bar{\theta }}_{0}\right)-a\left({\bar{\theta }}_{0}-\Delta /2\right) & a\left({\bar{\theta }}_{0}+\Delta /2\right)-a\left({\bar{\theta }}_{0}\right) \end{array}\right]$ to construct the smallest flat ellipsoid. Since the mismatch vector has been estimated (26) and emerged as the scaled projection vector of the presumed ASV onto the noise subspace, we now suppose that the *P* is updated with the projection vector $E_{n}E_{n}^{H}\bar{a}$. Then, to initialize *P*_1_ (corresponding to the first iteration), we make use of the projection vector $E_{n}E_{n}^{H}\bar{a}$ in tandem with *P*_small_ to construct the *P*_1_ with three columns in the first iteration:

(30) $$\begin{array}{c} P_{1}=\left[\begin{array}{ccc} a\left({\bar{\theta }}_{0}\right)-a\left({\bar{\theta }}_{0}-\frac{\Delta }{2}\right) & a\left({\bar{\theta }}_{0}+\frac{\Delta }{2}\right)-a\left({\bar{\theta }}_{0}\right) & E_{n}E_{n}^{H}\bar{a} \end{array}\right]. \end{array}$$

Then the optimal solution can be solved by substituting *P*_1_ into (11). Similar to Section 4.2, at the *i*th (*i* > 1) iteration, the center of the ellipsoid is updated with the previously estimated ASV. However, the corresponding *P*_*i*_ is composed of just two columns:

(31) $$\begin{array}{c} P_{i}=\left[\begin{array}{cc} E_{n}E_{n}^{H}{\hat{a}}_{{i-2}_{}}^{e} & E_{n}E_{n}^{H}{\hat{a}}_{{i-1}_{}}^{e} \end{array}\right]. \end{array}$$

Besides, the stopping condition is set as

(32) $$\begin{array}{c} \left\|E_{n}E_{n}^{H}{\hat{a}}_{i}\right\|\leq \eta , \end{array}$$

where *η* denotes the judgment threshold to check for the convergence. Also, *η* can be assigned a small value, similar to *ζ*. We summarize the concrete steps of the proposed algorithm below. Let ${\hat{a}}_{i}^{s}$ and ${\hat{a}}_{i}^{e}$ denote the estimated ASVs corresponding to the sphere set and ellipsoid set at *i*th iteration, respectively.

(i) Obtain the *E*_*n*_ through eigendecomposition of $\hat{R}$(8) and let ${\hat{a}}_{0}=\bar{a}$.
(ii) *For sphere*,
   at *i*th iteration, estimate the mismatch amount *ε*_*g*_^*i*^ using (20) and (21) and then calculate ${\hat{a}}_{i}^{s}$ by ${\left\|{\left(I+\lambda \hat{R}\right)}^{-1}{\hat{a}}_{i-1}\right\|}^{2}=\varepsilon_{g}^{i}$ and ${\hat{a}}_{i}^{s}=\bar{a}-{\left(I+\lambda \hat{R}\right)}^{-1}\bar{a}$.
 
*For ellipsoid*,
   at *i* = 1, initialize *P*_1_ as $P_{1}=\left[\begin{array}{ccc} \bar{a}-a_{\inf} & a_{\sup}-\bar{a} & E_{n}E_{n}^{H}\bar{a} \end{array}\right]$;
   when *i* > 1, $P_{i}=\left[\begin{array}{cc} E_{n}E_{n}^{H}{\hat{a}}_{i-2} & E_{n}E_{n}^{H}{\hat{a}}_{i-1} \end{array}\right]$;
   calculate ${\hat{a}}_{i}^{e}=\bar{a}+P_{i}\hat{u}$ by $\hat{u}=-{\left(\tilde{R}+\tilde{\lambda }I\right)}^{-1}\bar{\tilde{a}}$ and (12).
(iii) Update the presumed ASV $\bar{a}=\sqrt{M}\left({\hat{a}}_{i}/\left\|{\hat{a}}_{i}\right\|\right)$ for both sphere and ellipsoid.
(iv) If the stopping conditions (28) and (32) are satisfied, the iteration is ended, and the ${\hat{a}}_{i}$ is achieved. If not, go to step (ii).

## 5. Simulation Results

Assume a uniform array with *M* = 10 isolated elements spaced a half-wavelength apart. The noise in array system is modeled as additive white Gaussian noise with zero mean and unit variance. For each scenario, three incident sources (one desired signal and two interferences) are assumed to be plane wave. The precise DOAs and INRs of two interferences are set to be [30°, 20 dB] and [−25°, 20 dB], respectively. In all simulations, the actual direction of desired signal is *θ*_*s*_ = 6°, but the presumed steering direction is $\bar{\theta }=0^{{}^{\circ}}$. There is a look direction error of 6° and the corresponding uncertainty region is given as $\left[\begin{array}{cc} -7^{{}^{\circ}} & 7^{{}^{\circ}} \end{array}\right]$ with the mismatch Δ*θ* = 14°. To test the performance of the proposed methodology, there are three other robust beamformers compared with the proposed methodology in terms of array factors and output SINR: (I) sample matrix inversion (SMI), (II) diagonal loading (DL), and (III) robust capon beamformer (RCB). Moreover, some critical parameters in the above beamformers are chosen as follows: the loading factor (LF) in (II) and the uncertainty level in (III) are given as *ε*_rcb_ = 8.5 [13] and LF = 5, respectively. In addition, the DOA estimation errors and the random element errors are considered together in the last simulation. Each sensor is assumed to be displaced from the original position and the ASV caused by the position displacement is modeled as

(33) $$\begin{array}{c} a_{e}=\left[\begin{array}{ccccc} 1 & \cdots & e^{-j\varphi \left(m-1\right)} & \cdots & e^{-j\varphi \left(M-1\right)} \end{array}\right], \end{array}$$

where *φ* = (2*π*(*d* + *δ*_*m*_)/*λ*)sin*θ*, *d* is the distance between the reference element and current element, and *δ*_*m*_ is the displacement.

**Figure 2:**
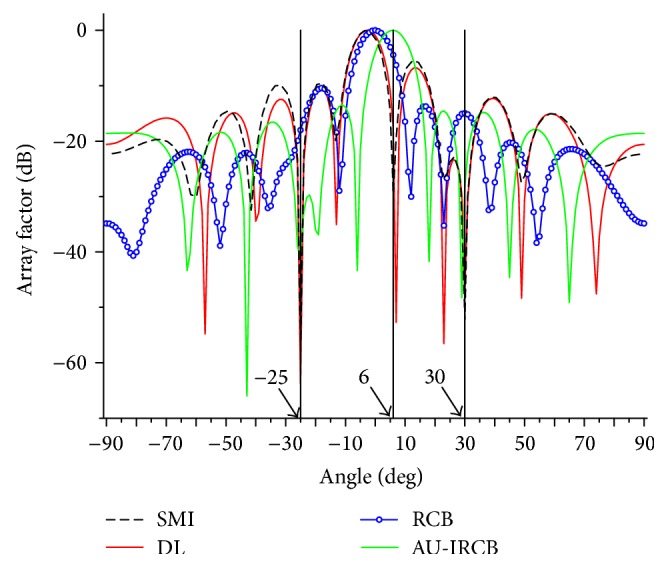
The comparison of array factors with spherical sets.

**Figure 3:**
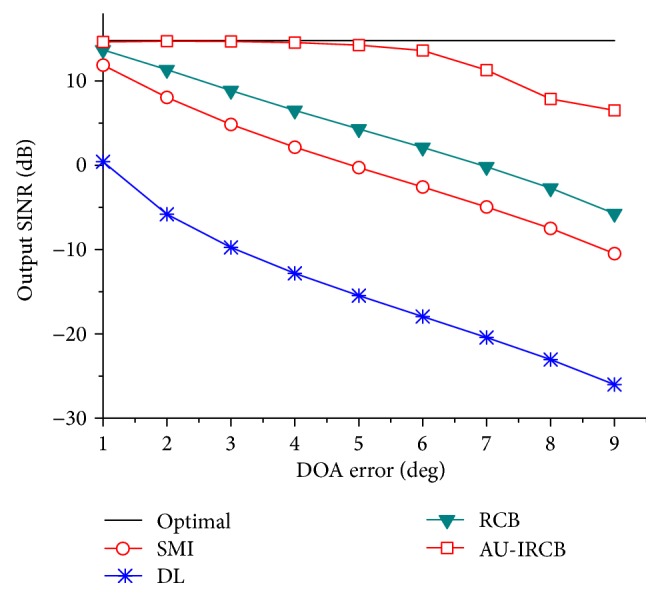
Output SINR versus DOA error.

In the first simulation, we compare the normalized array factors of the four beamformers. The numbers of snapshots and SNR of desired signal are fixed to be 200 and 5 dB, respectively.  Figure 2 shows that the main lobes of the SMI, DL, and RCB point to the presumed direction instead of the actual direction, while the proposed direction exactly and form nulls in the direction of −25° and 30°. The second example is operated with the same settings as example (I) to evaluate the output performance versus look direction error $\theta -\bar{\theta }$, which is varied from 1° to 9°. And the numbers of snapshots and input SNR are also set to be 200 and 5 dB, respectively. In Figure 3, the iterative methods acquire higher output SINR than others, especially in the case of large mismatch. Accordingly, the sensitivity of array to the look direction uncertainty is lowered and more robustness is provided by the Au-IRCB.

**Figure 4:**
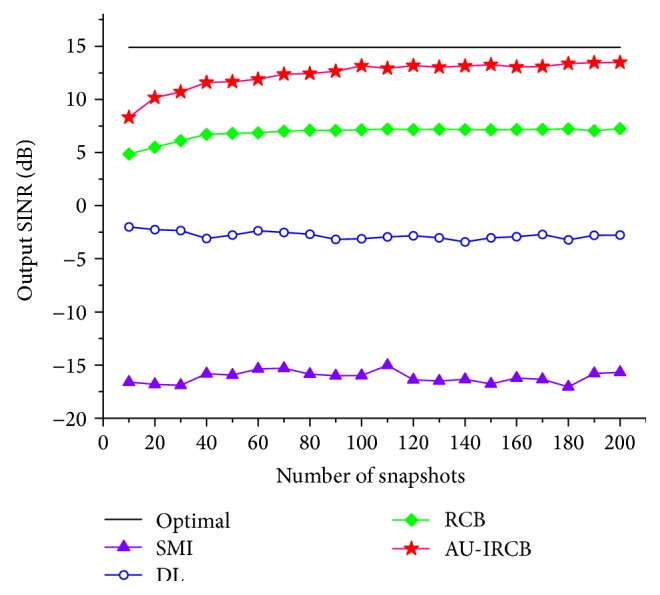
Output SINR versus snapshots.

The next two simulations concern the output SINR versus snap and SNR.  Figure 4 shows the output SINR of the four BFs versus the numbers of snapshots with the SNR selected as 5 dB. We vary the snap number from 20 to 200.

**Figure 5:**
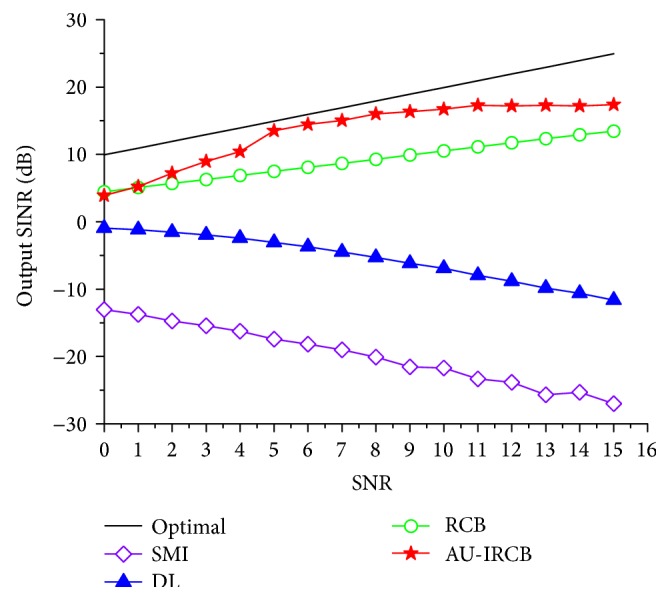
Output SINR against SNRs of the SOI.

It is observed that the AU-IRCB still maintain higher output than the other beamformers, even when the snap number is small. In the fourth example, the output performance of the same techniques versus SNR is illustrated in Figure 5 and the snap number is 200. One thing to note is that the performance of RCB is similar to the proposed method in the low SNR case; this imperfection is caused by the subspace overlapping.

**Figure 6:**
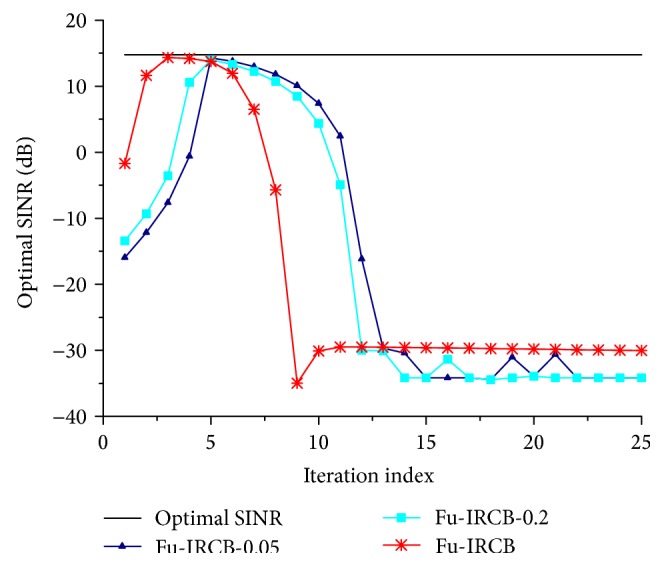
Output SINR versus iteration index.

In the fifth simulation, the convergence characteristic of Au-IRCB is tested to compare with the Fu-IRCB [17]. *ε*_fu_rcb_ is set to be 0.05 and 0.2, respectively. To observe the comparison intuitively, the stopping conditions are removed in Au-IRCB and Fu-IRCB.  Figure 6 shows that just 3 iterations are consumed by AU-IRCB before reaching the optimal state. And yet, the Fu-IRCB needs 6 iterations.

**Figure 7:**
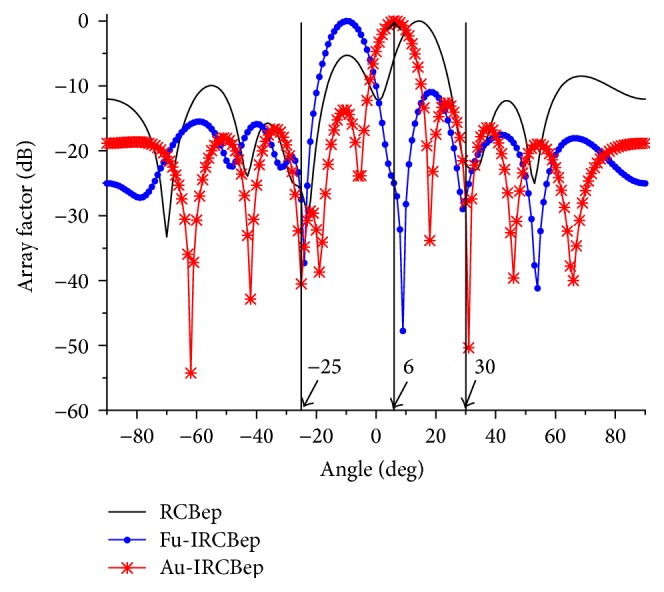
The comparison of array factors with ellipsoid sets.

In the last simulation, the DOA error and element displacement are considered simultaneously. The element position displacement is set to be distributed uniformly in the interval, $\delta_{m}\sim \left[\begin{array}{cc} -0.05 & 0.05 \end{array}\right]$, measured in wavelength. And the other conditions are the same with simulation (I). To verify the effectiveness of the new ellipsoid set, the robust beamformer with smallest flat ellipsoid uncertainty set (RCBep) [15] and the Fu-IRCB with fixed ellipsoid set (Fu-IRCBep) [17] are demonstrated to be compared with Au-IRCB using the new adaptively updated flat ellipsoid sets (Au-IRCBep) in the form of normalized array factor. In [15], Lorenz and Boyd propose an estimation method to calculate the smallest ellipsoid uncertainty sets. We rewrite the process as follows:

(34) $$\begin{array}{c} \bar{a}=\frac{1}{N}\overset{N}{\underset{i=1}{\sum }}a\left(\theta_{i}\right),\\ \alpha =\sup\left\{{\left(a\left(\theta_{i}\right)-\bar{a}\right)}^{H}P^{-1}\left(a\left(\theta_{i}\right)-\bar{a}\right)\right\},\\ P=\frac{1}{\alpha N}\overset{K}{\underset{i=1}{\sum }}\left(a\left(\theta_{i}\right)-\bar{a}\right){\left(a\left(\theta_{i}\right)-\bar{a}\right)}^{H}, \end{array}$$

where *N* is the number of the equally spaced samples in the interval $\left[\begin{array}{cc} {\bar{\theta }}_{0}-\Delta \theta /2 & {\bar{\theta }}_{0}+\Delta \theta /2 \end{array}\right]$ and *θ*_*i*_ = *θ*_inf⁡_ + (−1/2 + (*i* − 1)/(*N* − 1))Δ*θ*. Here, Δ*θ* = 14° and *N* = 1000. As considered in [17], the initial upper bound of *u* in the common ellipsoid sets is replaced by a fixed variable *ε*_*f*_ ≤ 1. The method searches the actual ASV iteratively until the stopping condition is satisfied. In this simulation, we choose *ε*_*f*_ = 0.02. The new ellipsoid set is shown as $\left\{Pu+\bar{a},{\left\|u\right\|}^{2}\leq \varepsilon_{f}\right\}$. In Figure 7, it can be seen that only Au-IRCBep shapes the beam pattern to focus the main beam on the direction of SOI and locate the one null just in the direction of −25° and the other in 30°. By contrast, the maximum array response of RCBep is not focused on the SOI. More severely, one null of the Fu-IRCBep lies in the vicinity of the desired direction. The two beamformers fail in the tackling with multiple mismatches.

## 6. Conclusion

A new robust beamformer is designed to iteratively search the array steering vector (ASV) of SOI to provide robustness against the large ASV mismatch. The proposed method updates the uncertainty levels adaptively based on a specific principle resulting from the subspace projection theory. This beamformer outperforms other methods in many aspects. We find that the estimated uncertain levels descend progressively and the number of iterations is reduced. On the other hand, the new AU-IRCB can be extended to tackle with more than one kind of mismatch using adaptively updated flat ellipsoid uncertainty sets. And more robustness is provided against the multiple defections by AU-IRCBep. Finally, the simulations results demonstrate the superiority of our method.
